# Research on the Rheological Characteristics and Aging Resistance of Asphalt Modified with Tourmaline

**DOI:** 10.3390/ma13010069

**Published:** 2019-12-21

**Authors:** Qunshan Ye, Wenzhuo Dong, Shipei Wang, Heng Li

**Affiliations:** 1 Key Laboratory of Road Structure and Material of Ministry of Transport (Changsha), Changsha University of Science and Technology, Changsha 410114, China; 2 Department of Traffic and Transportation Engineering, Changsha University of Science and Technology, Changsha 410114, China; dwz@stu.csust.edu.cn (W.D.); wangshipei@stu.csust.edu.cn (S.W.); hengli@stu.csust.edu.cn (H.L.)

**Keywords:** modified asphalt, tourmaline, negative ions treated, rheological property, aging resistance

## Abstract

Tourmaline modified asphalt (TMA) binders were prepared with different modifier types and contents in this research. The routine properties, rheological properties, and aging resistance were evaluated to research the function of tourmaline on the performances of asphalt binders. Test results show that the storage stability can be improved significantly by the smaller particle size and negative-ion treated surface of tourmaline modifier. It indicates that the stiffness and rutting-resistance of TMA binder can be enhanced significantly, and the elastic component of the viscoelastic characteristic can also be increased. Moreover, the complex viscosity and the Zero Shear Viscosity (ZSV) values of tourmaline modified asphalt are increased within the test frequency range, which results in the improvement of deformation resistance of tourmaline modified asphalt. When mixed with asphalt, the tourmaline modifier maintains a two-phase structure, which results in the good rheological property for tourmaline modified asphalt.

## 1. Introduction

A low-carbon economy has attracted more and more attention all over the world. Meanwhile, road engineers are paying attention to low-carbon materials, hoping to build green projects through low-carbon approaches [1,2,3]. Low-carbon engineering materials generally represent those engineering materials that can cut carbon dioxide emissions further. With the efforts of researchers, the scope of low carbon materials is expanding now. It usually means those kinds of materials which are capable of decreasing the carbon emissions as well as saving resources throughout the construction and service life [4]. Many researchers and engineers around the world are engaged in the research and development of advanced road materials, which can improve the performances and prolong the service life for asphalt pavement [5,6,7,8,9,10,11].

Tourmaline is an inorganic material with piezoelectric, thermoelectric, negative ion disengagement, and other characteristics [12,13,14,15,16]. Tourmaline has the environmental protection function because of its characteristics. For example, it can absorb harmful gases in the air to protect the environment. At the same time, it can save energy and resources in the production process. Furthermore, tourmaline asphalt can convert the energy attached to the vehicle into electricity by the hot spot effect and piezoelectric effect, which results in the decrease of temperature for asphalt pavement [17,18].

Wang et al. prepared tourmaline modified asphalt mixtures with ultra-fine powders and focused on assessing the impact of the materials on the environment [19,20,21]. The effects of inorganic fine powder on the performance of asphalt binders were studied [22]. The influence of calcium carbide modifier on the performances of pavement was emphatically studied [23,24]. The effects of tourmaline on asphalt density, dynamic stability, and flow value were analyzed, and the micro-mechanism of tourmaline modified asphalt was also studied [25,26,27,28]. Organic tourmaline exhibits excellent storage stability due to its reduced surface polarity, which pioneer a new way for the development of mineral and copolymer composites [29]. Çelikbilek et al. evaluated the performances of Tourmaline modified asphalt (TMA) mixture by the means of Grey multi-criteria evaluation model. It was indicated that the model had good reliability and practicability in TMA mixture, which can provide a new theoretical basis for the widely application of TMA [30].

The utilization of tourmaline in relative fields has become more and more popular. However, the comprehensive research on the properties of TMA and its mixture is still limited. The main work of this research is to study the properties of TMA under different modifier types and contents. The influences of tourmaline on storage stability, rheological characteristics, aging resistance, and microscope structure of TMA are all analyzed by various experiment methods.

## 2. Raw Materials and Preparation Process

### 2.1. Raw Materials

The bitumen used in the study was provided by Hunan Baoli Asphalt Co. Ltd. (Baoli, China). Properties of original asphalt are listed in the Table 1.

The chemical properties of tourmaline are shown in Table 2. Tourmaline modifiers used in this research are three types, tourmaline powder of 325 meshes (T1), tourmaline powder of 3000 meshes (T2), and Negative ions treated tourmaline powder of 325 meshes (T3). The content of modifier is 15%, 18%, and 21% by weight, respectively.

### 2.2. Preparation Process of TMA

The temperature in the oven was set to 150 °C in advance, the asphalt was placed in the oven for more than 1 h to make the asphalt liquid. The asphalt was taken out and placed in an electric furnace to maintain the temperature and stirred at a constant speed.

The dried tourmaline powder was weighed and slowly breathed into the original asphalt in the molten state in batches. After all the powders were added, they were stirred by hand for 10 min to keep the asphalt temperature from exceeding 170 °C. The pre-dispersed tourmaline modified asphalt was sheared by three steps. In the first step, the shearing speed was adjusted to 1000 rpm/min and continued for 10 min. The shearing speed was 5000 rpm/min during the second step and mixing for 30 min. Finally, the rotation speed was reduced to 1000 rpm/min for 10 min to overflow the air in the TMA.

### 2.3. Storage Stability Test

The original asphalt with a weight of 50 g was firstly placed into a 2.5-cm diameter aluminum tube. It was placed vertically for 48 h, heated to 163 ± 5 °C, and then placed immediately in to a refrigerator and cooled for 4 h. Finally, the hardened specimen was cut into three segments, each of the same length. Storage stability is capable of being evaluated by comparing the softening points of top and bottom samples. The smaller the difference between softening points, the better the storage stability.

### 2.4. Dynamic Mechanical Analysis

A dynamic shear rheometer (DSR) was used for the oscillation test. It applies oscillatory shear stress and strain to the asphalt sample sandwiched between parallel plates at different loading frequencies and temperatures.

The temperature sweep test frequency was set as 10 rad/s and a variable strain application temperature in the range of 45 to 70 °C. Approximately 1.0 g of the sample was placed on lower plate. After the sample is heated and melted, the parallel plate drops in operation, so that the sample is in close contact with the parallel team, and then the overflow part of the sample is built. The last clearance is 1.0 mm. The sample test temperature reaches 45 °C and can be kept warm for 10 min before the test begins. The temperature increment is set to 2 °C per minute, Strain control value γ = 12%. Various viscoelastic indices are automatically collected, including complex modulus ($G^{*}$) and phase angle (δ).

The frequency sweep test was applied at 60 ± 0.2 °C and the test frequency in a range of 0.1 to 100 rad/s, Strain control value γ = 1%. All samples should have been kept at the test temperature for 10 min, then used a scan frequency from fast to slow.

### 2.5. Bending Beam Rheometer (BBR) Test

The tests were conducted according to ASTM (American Society of Testing and Materials) D6648 to obtain an *S*-value and an m-value for various binders. The specimen is cylindrical, the specific size is 101.6 × 6.4 × 12.7 mm, and the test temperatures were −12 and −18 °C. The load on the specimen was 980 ± 50 mN. The test time was 60 s with an interval of 0.5 s. An asphalt binder of each type was repeated more than twice.

### 2.6. Aging Procedures

Short-term (production stage) aging test of various asphalt binders was processed by the rolling thin film oven (RTFO) test, and long-term (5–7 years) aging test of various asphalt binders was operated by the pressure aging vessel (PAV) test. The RTFO test was carried out by the AASHTO (The American Association of State Highway Transportation Officials) T240 [31] standard method at the standard temperature of 165 °C for 85 min. The PAV test was carried out according to AASHTO R28 standard operating procedure [32]. The test temperature was 100 °C, the air pressure was set to 2.1 MPa, and the test lasted for 20 h.

### 2.7. Scanning Electron Microscopy (SEM) Test

The microscopic morphology of TMA and the distribution of the modifier tourmaline powder in asphalt were observed by SEM to determine the interaction between tourmaline and the matrix asphalt. The instrument used in the test was a SUPRA55 high-performance field emission electron microscope produced by Zeiss (Oberkochen, Germany). The maximum voltage was 30 kv, the magnification was 12–1,000,000 times, and the maximum resolution was about 2.0 nm.

## 3. Results and Discussion

### 3.1. Storage Stability of TMA

The high-speed shear melting method of tourmaline modified asphalt is a mechanical method of mixing tourmaline powder with original asphalt. The TMAs prepared by this procedure are prone to separation of tourmaline and asphalt during storage, transportation, and construction. Such as tourmaline powder sinking, suspension rate reduction, etc.

**Figure 1:**
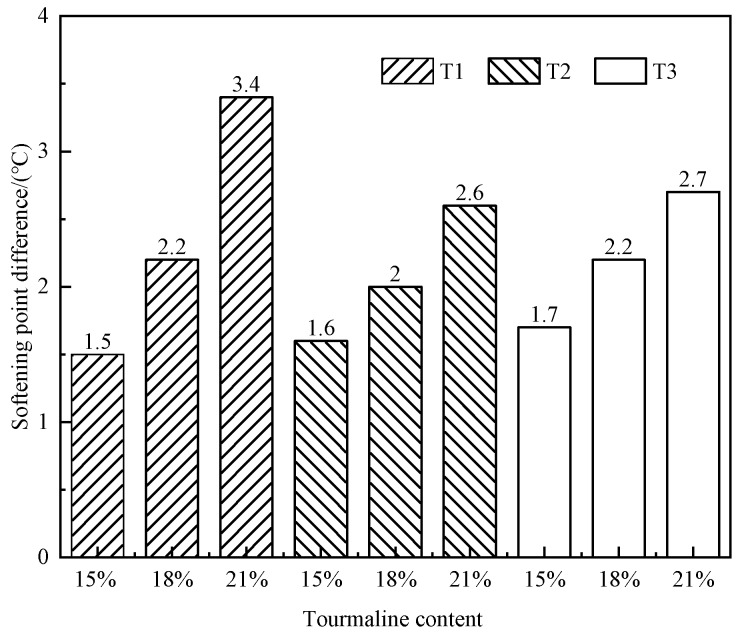
Storage stability of tourmaline modified asphalt (TMA) with different types and contents.

The temperature difference of the softening point of each TMA gradually increases with the increase of the amount of modifier, which demonstrates that the larger the content of tourmaline, the lower the compatibility of the modifier and the original asphalt. As shown in the Figure 1, it can be concluded that the TMA with the dosage lower than 18% has good storage stability. Furthermore, the storage stability of TMA is also affected by the particle size and surface of modifiers. The storage stability is strengthened significantly by tourmaline with smaller particle size and negative ions treated surface.

### 3.2. Rheological Properties of TMA at High Temperature

Viscoelastic characteristic of asphalt is affected by the environment temperature. It is viscosity when the temperature is high and elasticity when the temperature is low. The most important effect of modifier on asphalt is to enhance the viscoelasticity of asphalt, because shear resistance at high temperature has a strong correlation with the modulus of asphalt. The $G^{*}$ can be reflected the strength of asphalt. Asphalt resistance to permanent set becomes stronger with the increase of $G^{*}$ and the δ refer to the elastic ratio of the elastic capacity during shearing.

**Figure 2:**
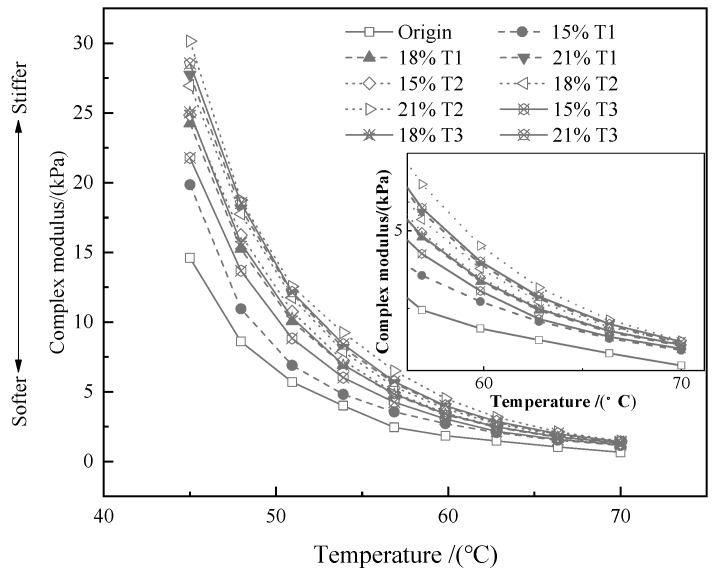
Effects of modifier types and contents on the $G^{*}$ of various binders.

**Figure 3:**
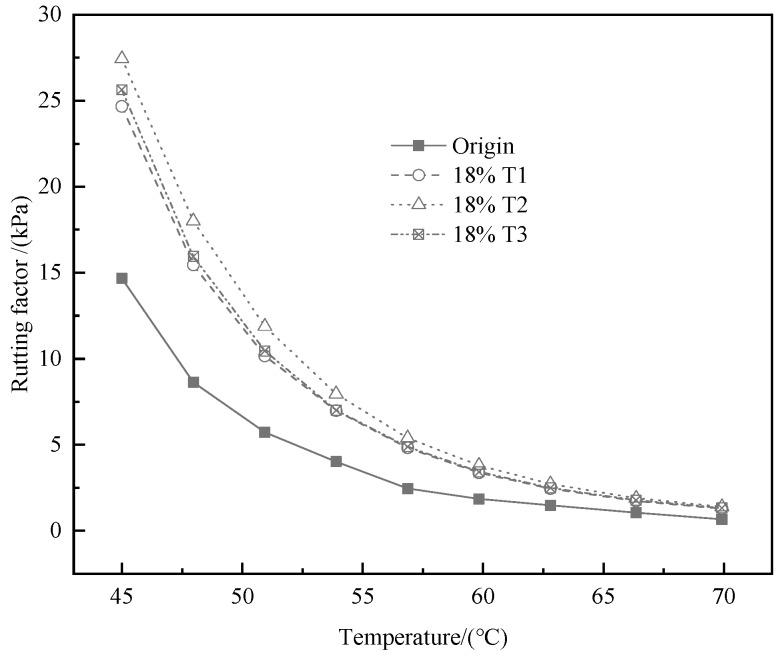
Effects of modifier types on the rutting factor of various binders.

**Figure 4:**
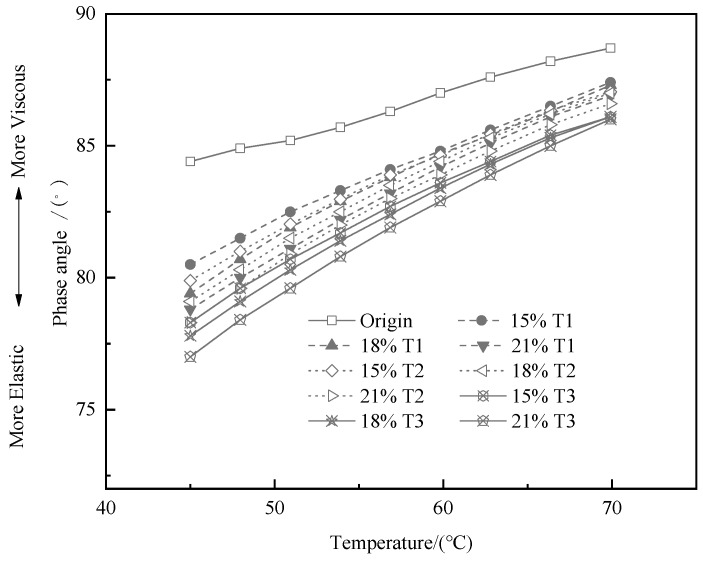
Effects of modifier types and contents on the δ of various binders.

The temperature sweep test results are known from Figure 2, Figure 3 and Figure 4. As shown in the Figure 2 and Figure 3, the $G^{*}$ and rutting factor of the TMA are higher than that of bitumen at test temperature. The value of $G^{*}$ increases with the addition of tourmaline. When the test temperature is between 45 and 70 °C, T2 modified asphalt has the strongest shear resistance because the $G^{*}$ is the largest. Similar information is capable of obtained from Figure 3 that the rutting factor of T2 modified asphalt is the largest, which implies the strongest resistance to permanent deformation. It means that the properties of asphalt on anti-rutting is able to be improved significantly by the addition of tourmaline modifiers, especially for the modifier with a smaller particle size [33].

The δ reflects the correspondence between the elastic deformation and the viscous deformation of the asphalt. The larger the δ, the worse the recovery ability after deformation for asphalt and its mixture. The development of the δ of the bitumen and TMA at test temperature can be seen in Figure 4. The δ of the bitumen and TMA increases with the temperature increasing, which signify that the viscous property is enhanced with the temperature that is rising gradually, while the elastic recovery capacity decreases. The δ value of TMA is lower than that of bitumen in the whole test temperature range, which reflects that the elastic recovery capacity at high temperature is strengthened by the utilization of tourmaline. At the same temperature, the bigger the content and fineness of tourmaline powder, the smaller the δ of TMA. Furthermore, the asphalt with the lowest δ is T3 modified asphalt, which reveals that the negative ions treated tourmaline behaviors better on the improvement of elasticity than that of untreated one.

**Figure 5:**
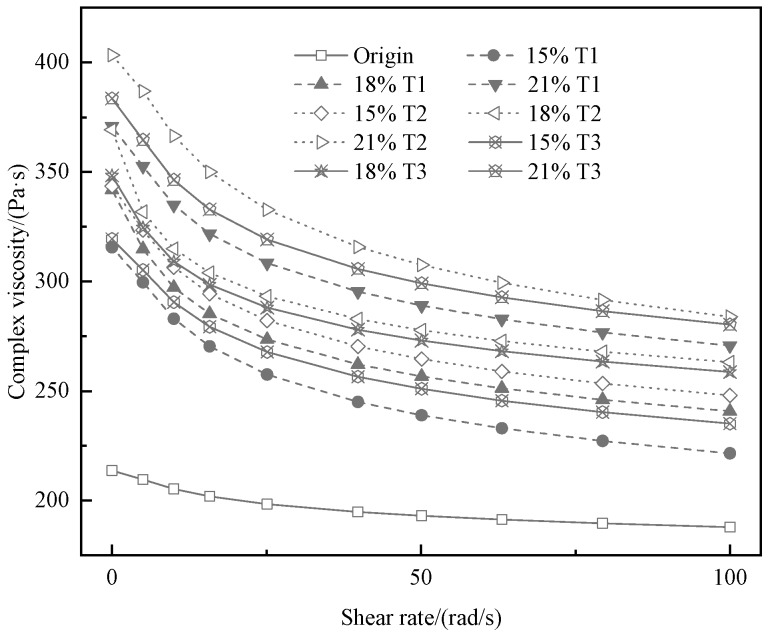
Complex viscosity of various asphalt binders at 60 °C.

**Figure 6:**
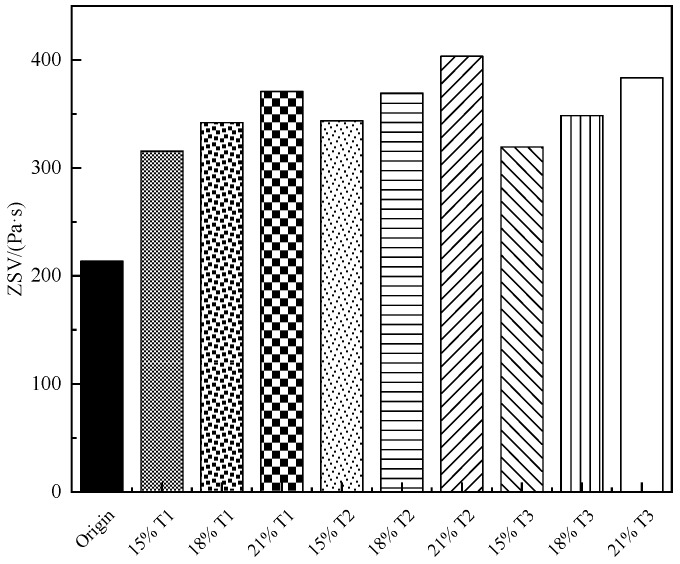
Zero shear viscosity (ZSV) of tourmaline modified asphalts of various types and contents.

Zero shear viscosity (ZSV) was used as an index to indicate that an ability to reflect asphalt used to resist permanent deformation in the frequency scanning test. The bigger the ZSV, the better the elasticity of asphalt and the smaller the permanent plastic deformation. The value of ZSV can be simulated by the Carreau model in this study. The outcome of the frequency sweep test is a simplified Carlo equation for polynomial fitting, which can be obtained from Figure 5. Figure 6 shows the ZSV values of various asphalt, including the original asphalt and TMA. It can be observed that the complex viscosity of the TMA is obviously higher than the complex viscosity of the original asphalt. The complex viscosity of the original and modified asphalt decreases with the faster shear frequency and increases with higher content of the tourmaline. The ZSV value increases as the tourmaline content increases. It can be observed that the elastic recovery capacity and the properties of asphalt to anti-rutting are improved by the T1, T2, and T3 [34]. When the same modifier content is adopted, the effect of improvement is the best for T2, followed by T3 and T1, which is consistent with temperature sweep test results mentioned above.

### 3.3. Rheological Characters of TMA at Low Temperature

**Figure 7:**
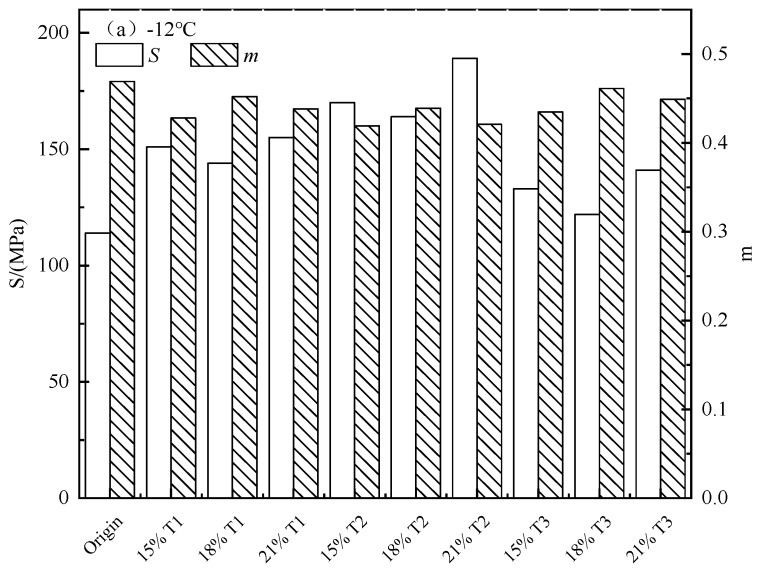

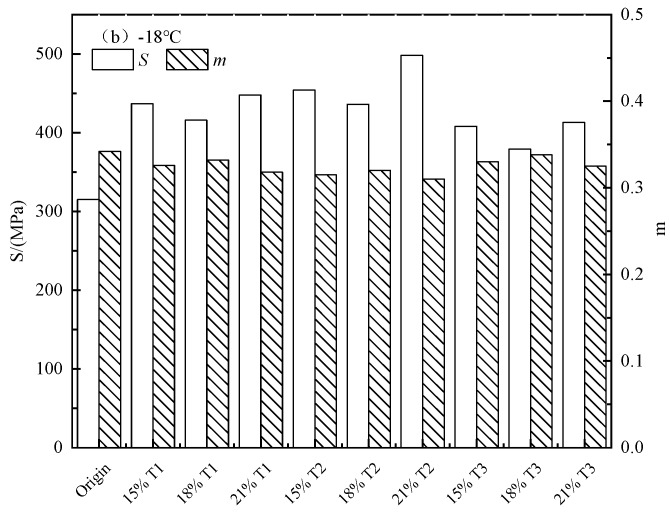
S and m for asphalt binders with different modifier content and type.

The BBR test outcome are shown in two part of Figure 7. It is capable of found that the *S*-value of TMA is significantly higher than that of original asphalt at test temperatures. Moreover, the creep rate m value is also reduced. It implies that the ability of asphalt binder to resist crack in low temperature will be weakened by the tourmaline modifier at low temperature. Furthermore, it can be observed that tourmaline modifier with a content of 18% has a better effect on the low temperature performance of modified asphalt. The rheological characters in the low temperature of modified asphalt are also capable to be affected by the modifier types, smaller particle size, and negative ions treatment shows better influences.

### 3.4. Aging Resistance of TMA

**Figure 8:**
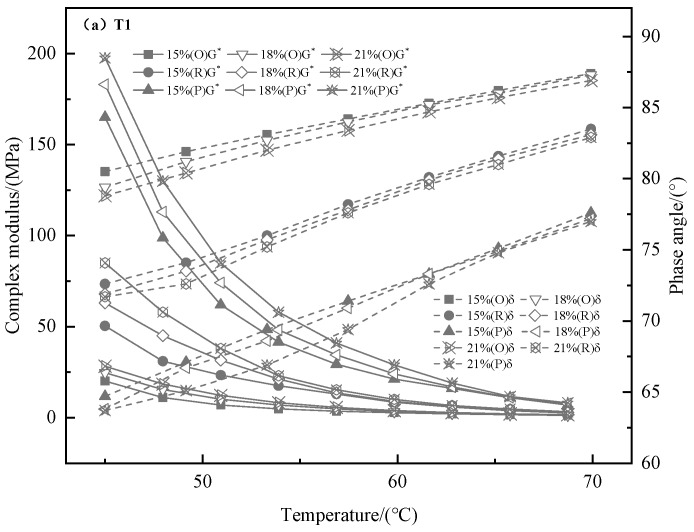

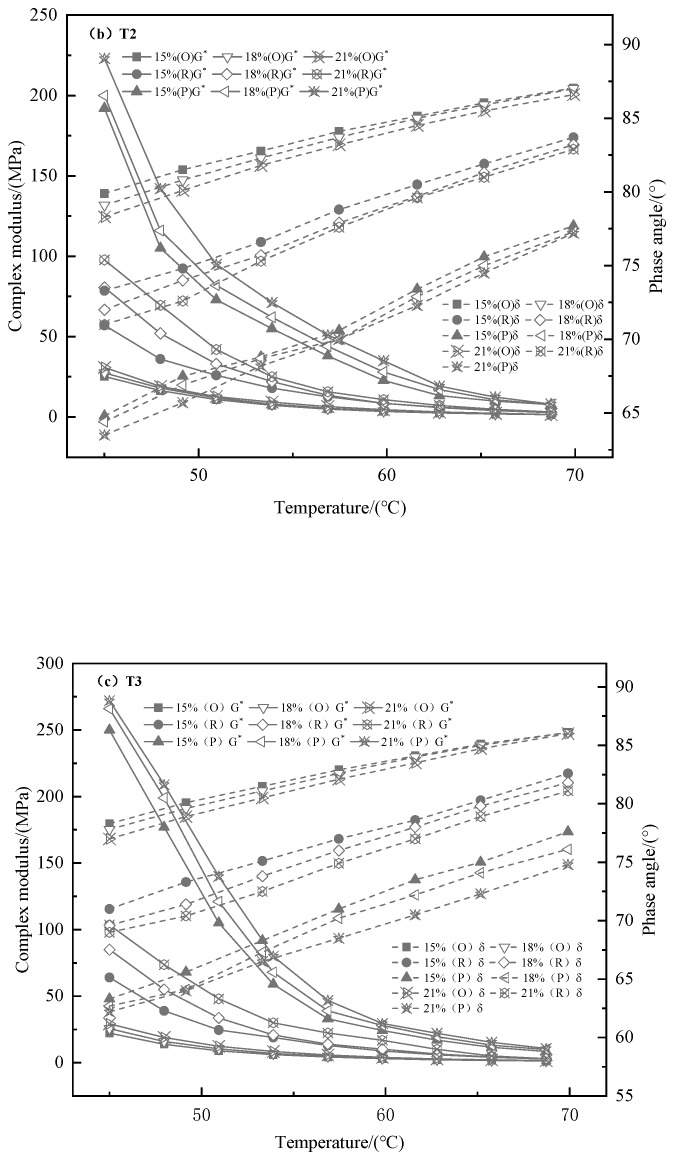
$G^{*}$ and for the TMA, including original and after aging test. (O—Origin, R—rolling thin film oven (RTFO), P—pressure aging vessel (PAV)).

**Figure 9:**
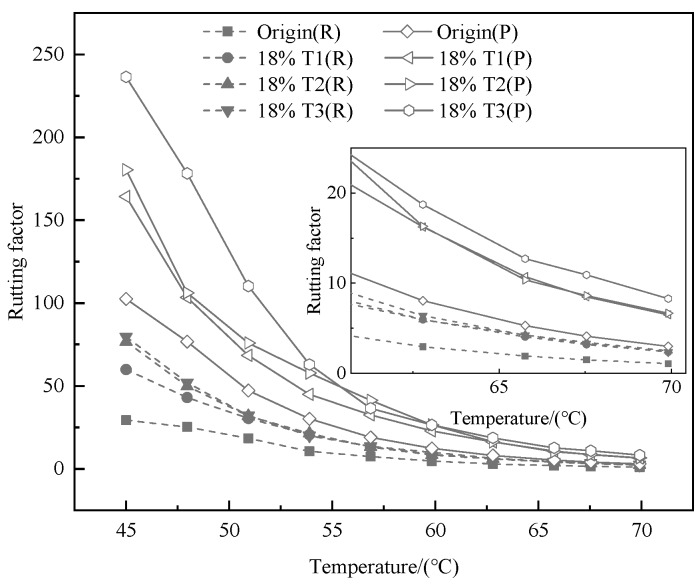
Rutting factor for the TMA, including original and after aging test. (R—RTFO, P—PAV).

The aging tests of the original asphalt and tourmaline modified asphalt were carried out by using a rotating film furnace and a pressure aging vessel. Figure 8 and Figure 9 reflect the rheological properties before and after aging procedure. After the original tourmaline modified asphalt were aged in different procedures, the $G^{*}$ increases and the δ of the modified asphalt decreases regularly. The reason may be that the asphalt binder becomes hard and brittle after the aging procedure, while the elasticity can also be enhanced. The rutting factor $G^{*}/\sin\delta$ of original asphalt and modified asphalt decreases rapidly with increasing temperature. Tourmaline modified asphalt has a higher rutting factor than original asphalt, which means that the properties of modified asphalt binder to combat shear deformation when the temperature is high can be enhanced by the addition of tourmaline modifier [35]. Moreover, the rutting factors for T3 modified asphalt binder after RTFO aging and PAV aging is always the best, which shown that the rheological characters of TMA binder can be promoted by negative ions treated method during two aging procedure, both RTFOT aging and PAV aging.

**Figure 10:**
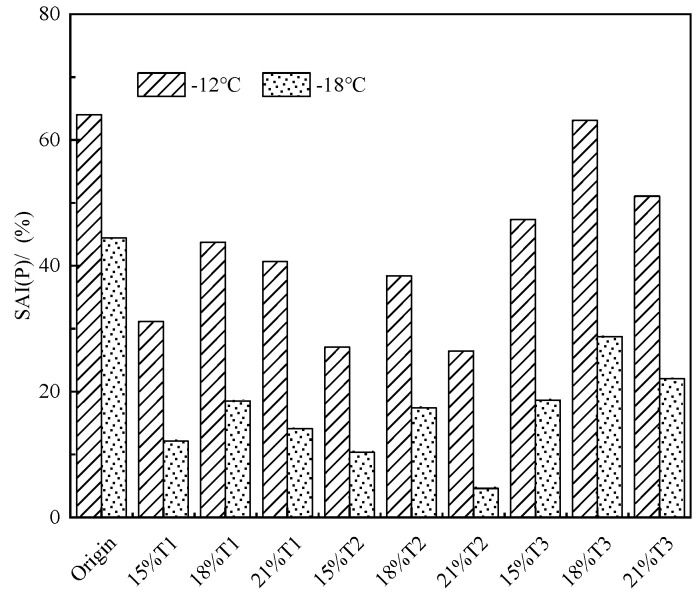
Stiffness aging index (*SAI*)(*P*) values for various asphalt binders.

Stiffness aging index (SAI) can be adopted to evaluate the properties of TMA to resist PAV aging. The index can be defined as following [36]:

(1) $$SAI\left(P\right)=\frac{S_{1}-S_{0}}{S_{0}},$$

in which, $S_{0}$ relate to the S-value of asphalt binder before PAV aging; $S_{1}$ means the S-value of asphalt binder after PAV aging. The lower SAI(P) value indicates better aging resistance for asphalt binder. The SAI(P) for various asphalt are capable to seen from Figure 10.

Figure 10 is shown that the SAI(P) of all TMA is smaller than that of original asphalt at −12 °C and −18 °C, which indicates that the ability of modified asphalt binders to resist aging can be enhanced by the addition of tourmaline modifiers. Effects of particle size of the modifier on the aging resistance can also be observed in this study. The particle size of the tourmaline modifier is smaller, the stronger the anti-aging property of the TMA. When the test temperatures were −12 °C and −18 °C, the SAI(P) of T2 modified asphalt binder is the lowest, which results in the best aging resistance for modified asphalt binder.

### 3.5. Result of SEM

**Figure 11:**
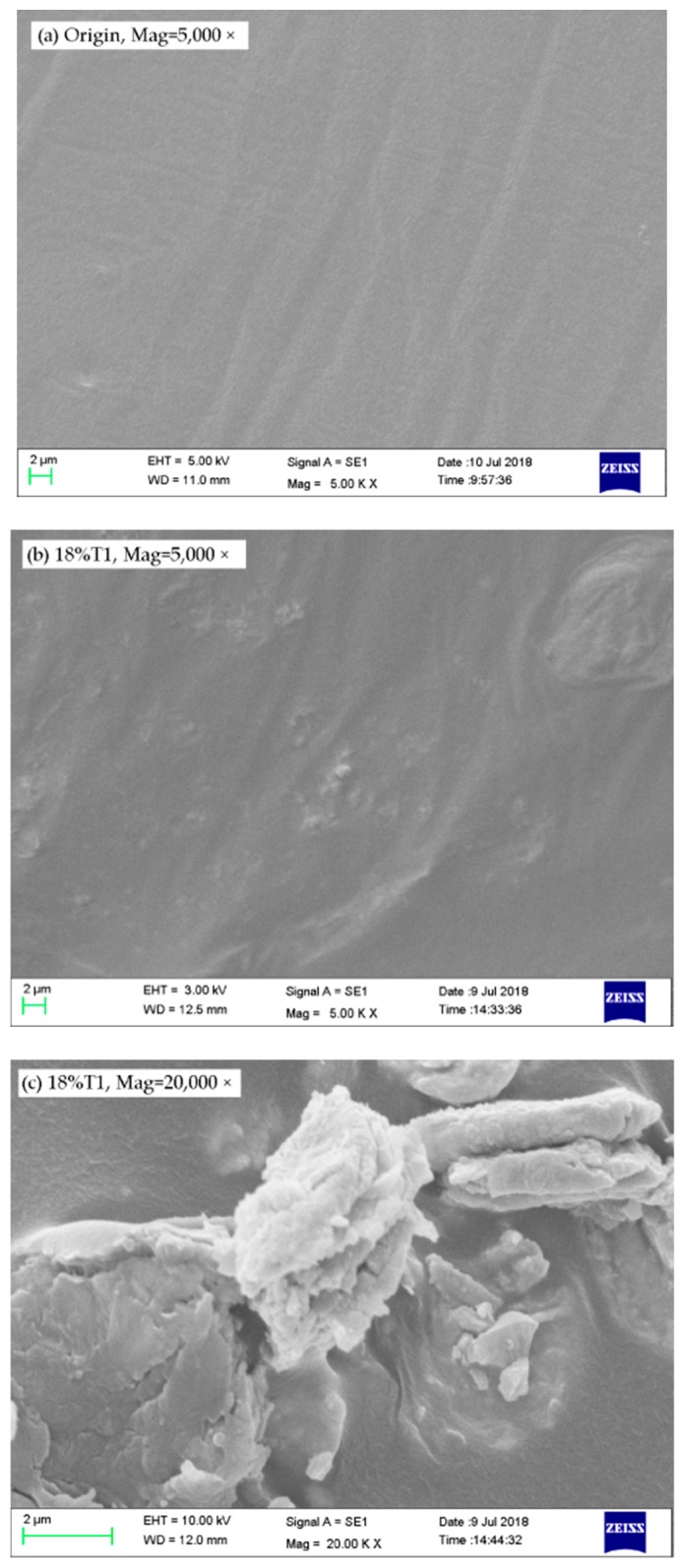
Images for original asphalt and T1 modified asphalt binder. (**a**) Original asphalt, magnification = 5000×; (**b**) 18% T1 modified asphalt, magnification = 5000×; (**c**) 18% T1 modified asphalt, magnification = 20000×.

The content of Figure 11 is the microscopic images for various asphalt binders. It is able to discover From Figure 11a,b, the surface of the matrix asphalt is dense and has silky wrinkles, and the structure can be considered to be completely uniform. The tourmaline is dispersed in different particles, and the particles are connected to the particles through the asphaltene to form a two-phase dispersion system. When mixed with asphalt, the tourmaline modifier maintains a two-phase structure. This structure determines the good rheological property of the TMA.

The microscopic morphology of the TMA is shown in Figure 11c. Due to the autogenously polarity of tourmaline, some fine particle groups adhere to the surface of large particles, and the large surface energy tends to shrink into an energy state. There is no coating around the tourmaline particles. Under the long-term action of the molecule, the interfacial transition layer should be formed from certain components of the asphalt. This also confirms the fact that there was no complicated chemical process between tourmaline and matrix asphalt. Instead, it is completely permeable and is directly wrapped by matrix asphalt. The tourmaline material is characterized by porosity, a rough surface and a large specific surface area, so it preferentially absorbs the light oil in the hot asphalt and the saturated portion of the asphalt to cause adsorption. The bitumen component penetrates and adsorbs inside the tourmaline and forms a thicker interfacial layer with itself. The interface layer is connected to two phases. It is a spatial region with a certain thickness at the boundary of the two phases. It transfers and buffers the surface viscosity and surface between the two phases. Pressure, surface tension, etc. It has important influence on the performance of TMA, especially the mechanical properties and the physical characters. The temperature sensitivity of tourmaline modified asphalt is weakened by thick interfacial layers. After the adsorption is completed, the saturated component and the aromatic component in the asphalt are relatively reduced, the colloid and asphaltene content are relatively increased, and the tourmaline and the asphalt are closely combined, and both have good cohesiveness and resistance to permanent deformation.

## 4. Conclusions

TMA was prepared by high-speed shearing instrument and the conventional properties, rheological characters, and the properties of anti-aging of TMA were studied by relevant indoor macroscopic tests. The following results were obtained:

(1) The storage stability of TMA binder was capable significantly improved by the smaller particle size and negative ions treated surface of the tourmaline modifier.
(2) The complex modulus ($G^{*}$) and $G^{*}/\sin\delta$ of asphalt binder were increased by the tourmaline modifier, which indicated the enhancement of the strength and rutting resistance for asphalt binders. The decrease of phase angle for modified asphalt binder reveals the increase of elastic components in viscoelastic characters of asphalt binder.
(3) The ZSV values of TMA binder were increased and the elastic recovery capacity and the properties of asphalt are used to resist rutting are enhanced by the tourmaline modifier.
(4) The low temperature rheological characters of modified asphalt is also can be deteriorated by the tourmaline to some extent. However, modifier with a smaller particle size and negative ion treated surfaces show better influences.
(5) The rheological properties of asphalt binder were improved by the tourmaline modifier, either after RTOFT or PAV. The asphalt binder modified with the smaller particle size of tourmaline has better aging resistance.
(6) The light oil and saturated portion of the asphalt can be absorbed by the tourmaline modifier. A thicker interfacial layer can be formed between tourmaline and asphalt, which results in the enhanced properties that can be used to resist permanent deformation.

## Figures and Tables

**Table 1:** Parameters of the original asphalt.

Properties	Value	Methods
Penetration (25 °C, 0.1 mm)	68.7	ASTM D-5
Softening point (°C)	47.3	ASTM D-36
Ductility (15 °C, cm)	150	ASTM D-113
Ductility (10 °C, cm)	86	ASTM D-113
Viscosity (60 °C, Pa·s)	298	ASTM D-4402

**Table 2:** Main chemical constituents of two tourmaline powders.

-	Content (%)								
-	SiO_2_	Al_2_O_3_	Fe_2_O_3_	B_2_O_3_	MgO	CaO	MnO	Na_2_O	FeO
tourmaline powder	35.75	31.15	8.48	8.82	4.50	6.32	1.20	0.91	1.30
negative ions treated	40.02	31.15	8.48	10.82	4.50	0.16	1.20	0.91	1.30
